# Nanomechanical Characterization of Vertical Nanopillars Using an MEMS-SPM Nano-Bending Testing Platform

**DOI:** 10.3390/s19204529

**Published:** 2019-10-18

**Authors:** Zhi Li, Sai Gao, Uwe Brand, Karla Hiller, Susann Hahn, Gerry Hamdana, Erwin Peiner, Helmut Wolff, Detlef Bergmann

**Affiliations:** 1 Physikalisch-Technische Bundesanstalt, Bundesallee 100, D-38116 Braunschweig, Germany; sai.gao@ptb.de (S.G.); Uwe.Brand@ptb.de (U.B.); helmut.wolff@ptb.de (H.W.); Detlef.Bergmann@ptb.de (D.B.); 2 Fakultät für Elektrotechnik und Informationstechnik, Zentrum für Mikrotechnologien Chemnitz, Technische Universität Chemnitz, Reichenhainer Straße 70, 09126 Chemnitz, Germany; karla.hiller@zfm.tu-chemnitz.de (K.H.); Susann.Hahn@zfm.tu-chemnitz.de (S.H.); 3 Institute of Semiconductor Technology (IHT) and Laboratory for Emerging Nanometrology (LENA), Technische Universität Braunschweig, 38106 Braunschweig, Germany; g.hamdana@tu-braunschweig.de (G.H.); e.peiner@tu-bs.de (E.P.)

**Keywords:** nanomechanical characterization, nanoindentation, nanomechanical properties, nanopillars, microelectromechanical system, scanning probe microscopy

## Abstract

Nanomechanical characterization of vertically aligned micro- and nanopillars plays an important role in quality control of pillar-based sensors and devices. A microelectromechanical system based scanning probe microscope (MEMS-SPM) has been developed for quantitative measurement of the bending stiffness of micro- and nanopillars with high aspect ratios. The MEMS-SPM exhibits large in-plane displacement with subnanometric resolution and medium probing force beyond 100 micro-Newtons. A proof-of-principle experimental setup using an MEMS-SPM prototype has been built to experimentally determine the in-plane bending stiffness of silicon nanopillars with an aspect ratio higher than 10. Comparison between the experimental results and the analytical and FEM evaluation has been demonstrated. Measurement uncertainty analysis indicates that this nano-bending system is able to determine the pillar bending stiffness with an uncertainty better than 5%, provided that the pillars’ stiffness is close to the suspending stiffness of the MEMS-SPM. The MEMS-SPM measurement setup is capable of on-chip quantitative nanomechanical characterization of pillar-like nano-objects fabricated out of different materials.

## 1. Introduction

Micro- and nanopillars made of different materials have found extensive applications in various scientific and industrial fields, including energy harvesting [1], energy storage in batteries [2], illumination [3], and micro- and nano-force sensing [4,5,6]. Rapid advances in fabrication technology [7] have already enabled sub-micro-pillars with an aspect-ratio (AR) much higher than 10 to be well produced. For the purpose of reliable applications of these pillar-based sensors and instruments, it is therefore demanded that the physical and nanomechanical properties of pillars should be quantitatively characterized. Especially in the case of force sensing at the nanoscale, quantitative determination of the in-plane bending stiffness of vertically aligned pillars has gained great interest.

Theoretically, the bending stiffness of micro- and nanopillars can be analytically estimated, once the geometrical parameters of the pillars are carefully determined, and the mechanical properties of pillars are known. However, such analytical approach usually shows large estimation deviation, due to several reasons, such as the imperfect nano-fabrication of the pillar geometry, deviating mechanical properties of nanomaterials at the sub-micrometer scale, and the difficulty in quantitative 3D measurement of the nanopillars’ geometry and topography.

As a result, experimental approaches [8] have gained much interest for nanomechanical characterization of micro- and nanopillars, including nanoindentation [9], lateral atomic force microscopy (AFM) [10], and AFM-based nano-bending tests [11]. The latter features a relatively straightforward measurement principle, only few requirements for sample preparation, and the capability for extraction of the overall mechanical properties of nanopillars made of complex materials and hybrid structures, and therefore has long been applied for qualitative characterization of nanopillars with very high ARs.

However, it is already known [12,13] that AFM nanomechanical measurements, particularly in the case of lateral bending testing, usually suffer from such essential problems as unavoidable cross-talk between lateral and vertical deflection of AFM cantilever in measurement, considerable nonlinearity in the case of large deflection of AFM probes, and limited lateral and normal testing force ranges. To calibrate the lateral stiffness of AFM probes with high accuracy, innovative calibration standards including microelectromechanical system (MEMS) based microforce sensors [14] are needed.

In this manuscript, our efforts for quantitatively determining the bending stiffness of high-AR pillars using a MEMS-based scanning probe microscope (MEMS-SPM) are presented. Design and numerical investigation of the MEMS nano-force transducer is detailed in Section 2. Mechanical characterization of the MEMS transducer and first results of nano-bending tests of silicon nanopillars are reported in Section 3.

## 2. Development of a MEMS Scanning Probe Microscope

To avoid the aforementioned drawbacks of conventional AFM nanomechanical measurements but to utilize the AFM probes’ main advantages to full capacity, an MEMS-SPM is developed in the Physikalisch-Technische Bundesanstalt (PTB Braunschweig), based on a MEMS nano-force transducer with post-assembled AFM tip to sense the tip–surface interaction with a high force sensitivity, and to further perform bending tests with a probing force up to 100 micro-Newtons.

### 2.1. MEMS-SPM Head

**Figure 1:**
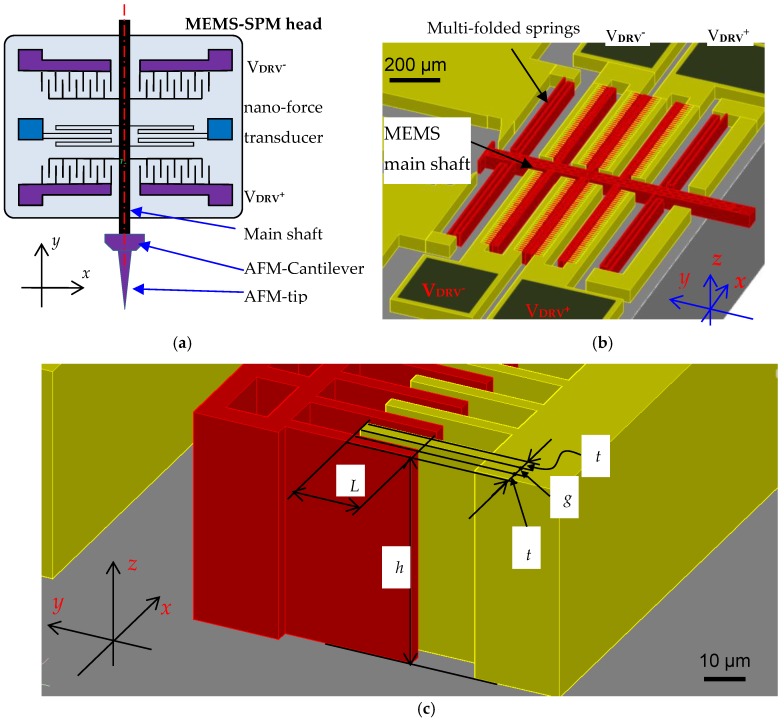
Design of a microelectromechanical system based scanning probe microscope (MEMS-SPM) for quantitative nanomechanical characterization of nanopillars: (**a**) Schematic diagram of the MEMS-SPM, where an atomic force microscopy (AFM) probe was glued onto the end of the shaft; (**b**) 3D-design of the MEMS nano-force transducer; (**c**) detailed view of the MEMS finger pairs, where *L* is the overlap length of the comb finger pairs, *h* is the vertical height of the fingers, *t* is the in-plane thickness of the fingers, and *g* is the gap of the comb pairs.

The fundamental principle of the MEMS nano-force transducer (or in short, MEMS-SPM head) is illustrated in Figure 1a. It typically consists of two sets of lateral electrostatic comb-drive actuators [15,16] in a differential configuration. The comb-drives have movable and fixed fingers with a typical in-plane thickness of *t* = 3 µm, and a typical gap of the finger pairs *g* = 3 µm. The out-of-plane height of the finger pairs (and also the suspending springs) *h* is selectable for prototyping. This differential design of the MEMS transducer is aimed not only for doubling the resolution for displacement sensing, but also for common-mode noise suppression.

The main shaft and moving part of the MEMS is supported by a group of suspending springs. It is already well known that a suspending system with multi-folded beam-like springs can not only exhibit a relatively small stiffness, but also good linearity for large displacements. Two pairs of springs are, therefore, employed in the MEMS mechanical system, as shown in Figure 1b.

The MEMS-SPM is intended to be used for surface profiling and nanomechanical measurement in contact mode. Under the consideration of the force sensitivity and measurement range of the MEMS-SPM, the stiffness of the MEMS force transducer along its moving axis (i.e., y-axis in Figure 1b, *k_y_*) is chosen to be ~13 N/m. 

A well-designed mechanical system of the MEMS ensures that its stiffnesses along the axes perpendicular to its moving axis (i.e., *k_x_*, *k_z_* in Figure 1b) are far larger than *k_y_*, so that the MEMS transducer is linearly guided in y-direction.

**Figure 2:**
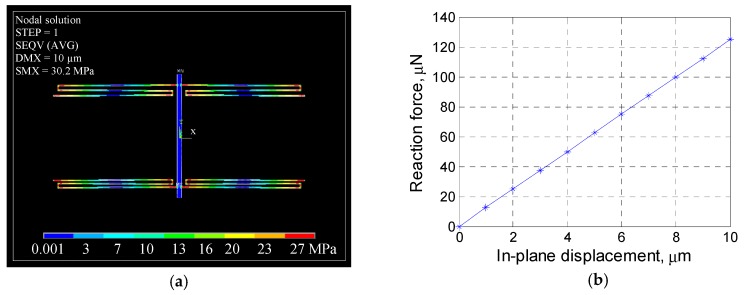
Finite element modelling (FEM) of the MEMS quasi-static performance: (**a**) In-plane (x–y plane) stress in the MEMS suspending system (here, DMS is the main shaft’s maximum in-plane displacement); (**b**) relationship between the MEMS reaction force (corresponding to the probing force) and the MEMS in-plane displacement.

The quasi-static performance of the MEMS mechanical system has been numerically investigated with the help of finite element modelling (FEM). For the MEMS springs with a vertical thickness *h* of 50 µm, as illustrated in Figure 2a, the maximum in-plane stress of the MEMS suspending system under its maximum in-plane displacement (10 µm) amounts to about 30 MPa, which is far less than the fracture strength of single-crystal silicon (>10 GPa) [17]. The corresponding reaction force is found to be 124.8 µN, indicating the stiffness of the MEMS to be 12.5 N/m.

### 2.2. Prototyping

The MEMS devices numerically designed in Section 2.1 have been fabricated using a deep reactive ion etching technique combined with a silicon–silicon bonding step, the so-called bonding-deep reactive ion etching (B-DRIE) [18]. By application of this B-DRIE technology, silicon micro-structures with an aspect ratio higher than 15 can be produced. Within this prototyping, the typical vertical thickness of micro-finger pairs and suspending beams is chosen to be *h* = 50 µm.

**Figure 3:**
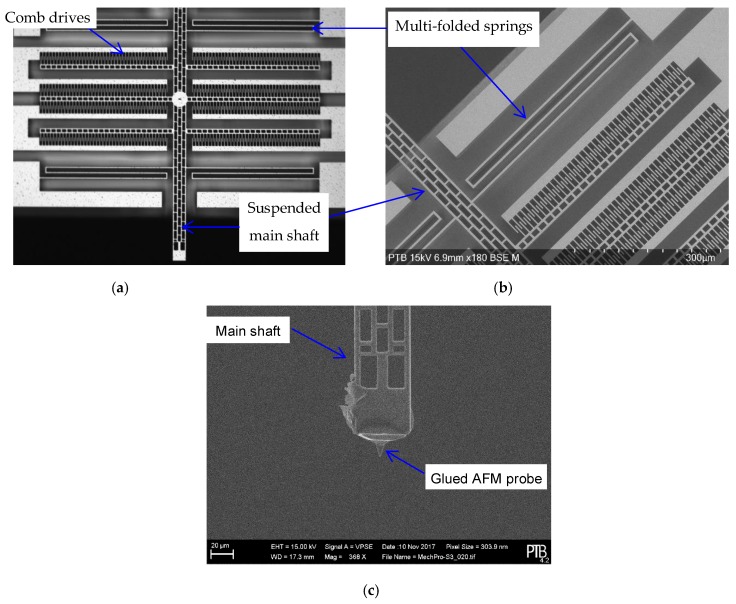
Prototyping of the MEMS-SPM using the bonding-deep reactive ion etching (B-DRIE) technology [16]: (**a**) Microscopic image of the MEMS nano-force transducer with folded springs and electrostatic comb-drive for force and displacement sensing; (**b**) SEM image of the MEMS nano-force transducer; (**c**) prototype of a MEMS-SPM with a glued AFM probe (the diamond coated tip height amounts to ~15 µm).

Figure 3a shows an optical overview of a MEMS SPM prototype, and Figure 3b shows a detailed SEM image of the prototype. For the purpose of surface sensing, an AFM probe is manually glued onto the main shaft of the MEMS transducer, as shown in Figure 3c.

Taking into consideration that the MEM-SPM should be able to perform nanomechanical measurement with the testing force up to ~100 µN, an AFM probe coated with diamond film is preferred. The tip rounding of the diamond coated probe amounts to about 100 nm.

Using the home-developed MEMS stiffness calibration system detailed in [18], the suspending stiffness of the MEMS force transducer is found to be 12.8 ± 0.5 N/m.

## 3. Nanomechanical Characterization of Nanopillars Using the MEMS-SPM

To demonstrate the capability of the MEMS-SPM prototype developed in Section 2, a proof-of-principle experimental setup has been built.

### 3.1. System Configuration

**Figure 4:**
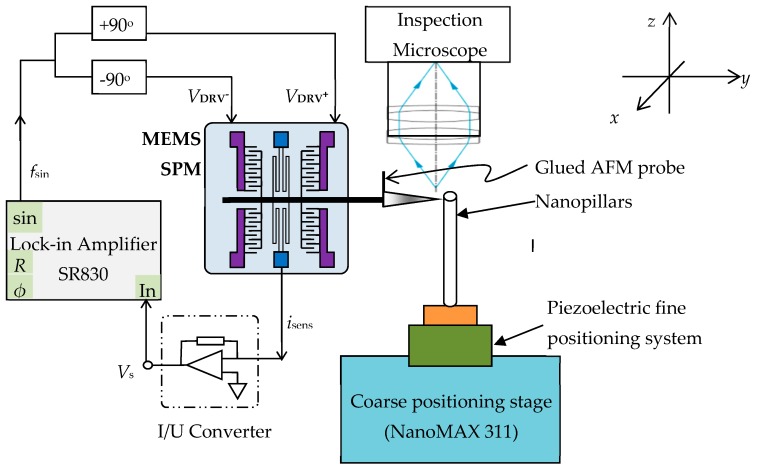
Experimental setup of the MEMS-SPM for nanomechanical measurements.

As illustrated in Figure 4, a three-axis micro-positioning stage combined with an integrated 3D closed-loop piezo-stage (NanoMAX 311, Thorlabs Inc., Newton, NJ, USA) is utilized to position and engage the specimen under test to the MEMS-SPM head. This nano-positioning stage equipped with three fine micrometers offers an *x*–*y*–*z* coarse positioning range up to 4 mm x 4 mm x 4 mm with micrometer resolution. The closed-loop piezo-stage exhibits a fine positioning range up to 20 µm for each axis, with a closed-loop resolution better than 5 nm.

The in-plane displacement of the MEMS main shaft together with the mounted AFM cantilever is measured using the capacitive sensing technique detailed in [12,19]. An excitation signal (*f*_sin_) coming from a lock-in amplifier (SR 830, Stanford Research Systems, sin) is firstly divided into two channels with a phase difference of 180°, and then applied to the MEMS electrodes *V*_DRV_^+^ and *V*_DRV_^–^ (see Figure 1b), respectively. The sensing current signal collected from the MEMS *i*_sens_ is converted into the voltage signal *V_s_* by means of a current-to-voltage converter, finally sent to the lock-in amplifier (In) for the extraction of the in-situ displacement (amplitude *R* and phase *ϕ*) of the MEMS main shaft.

The excitation signal has a frequency *f_m_* = 100 kHz, which is far higher than the resonance frequency of the MEMS (*f*_MEMS_ = 3.5 kHz). Therefore, no cross-talk between the displacement sensing system and the MEMS can arise.

Previous experiments [12,19] have already verified that a displacement resolution of 0.2 nm and a nonlinearity of 0.25‰ can be achieved with this capacitive readout system.

For convenience, a home-built inspection microscope has been utilized for optical inspection of the pillar samples and for coarse positioning of the MEMS-SPM tip on the sample.

### 3.2. Sample Preparation

**Figure 5:**
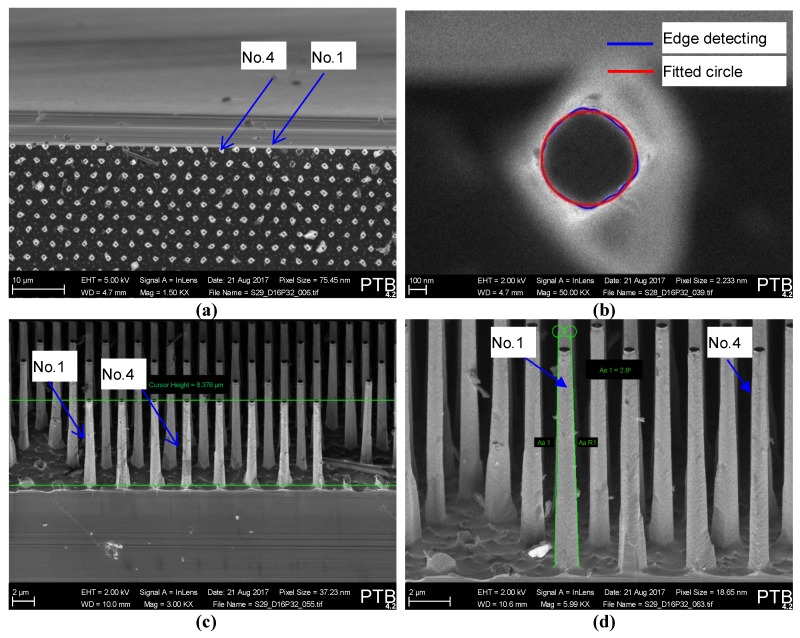
SEM measurement of the geometrical dimensions of silicon nanopillars: (**a**) SEM overview of the silicon nanopillars (pillar’s nominal diameter Ø*D* = 780 nm, height *H* = 11.0 µm); (**b**) determination of the top diameter of the nanopillar No. 1 using SEM; (**c**) SEM determination of the average height of silicon nanopillars (Note: The sample is tilted by 45°); (**d**) determination of the tapering angle of nanopillars by SEM (Note: The sample is tilted by 65°).

**Figure 6:**
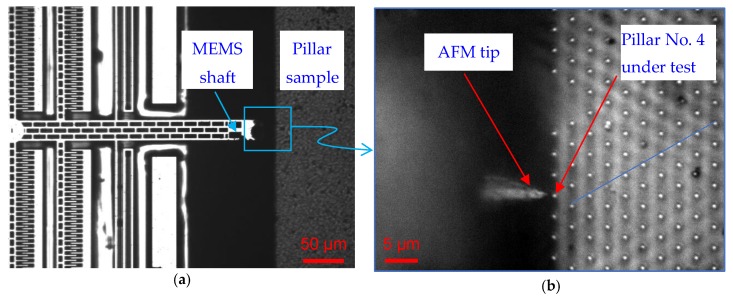
Nanopillars under measurement: (**a**) Auxiliary optical microscope (5x, NA = 0.13) image of the MEMS-SPM and the silicon nanopillar sample (top view); (**b**) detailed view of the AFM tip and the pillars under nanomechanical measurement with an Olympus objective (80x, NA = 0.75).

The mechanical properties of silicon nanopillars fabricated by templated-nanoparticle–array lithography followed by deep reactive ion etching at cryogenic temperature (cryoDRIE) [20] have been investigated using the aforementioned experimental setup. The silicon nanopillars have a nominal lateral pitch of 3.2 µm, with a nominal height of 11 µm and nominal diameter of 780 µm. Careful sample preparation has ensured that plenty of nanopillars exist close to the edge of the silicon substrate, in order to access the pillars from the side for bending experiments (see Figure 5a and Figure 6b).

For the purpose of analytical estimation of the pillars’ bending stiffness, the geometrical dimensions of the nanopillars have been determined by means of SEM imaging. As detailed in Figure 5c, the silicon pillars show a tapered 3D form. The top diameter *D_t_* of the pillars has to be measured one by one, as shown in Figure 5b. After the sample is tilted to 45°, the averaged pillar height is measured, as illustrated in Figure 5c, and the taper angle of each pillar is also determined one by one.

It can be seen from Figure 5c,d that, in comparison with others, the pillars No. 1 and No. 4 have relatively less shape imperfections. Especially the 3D shape of the pillar No. 4 can be well modelled by a tapered cylinder. In the proof-of-principle measurement, these two pillars will be characterized with the nano-bending measurement system. For comparison, the measured geometrical dimensions of the nanopillars No. 1 and 4 are summarized in Table 1. The bottom diameter *D_b_* of the tapered pillars is calculated as *D_b_* = *D_t_* + 2*H*·tan*γ_z_* with *γ_z_* as the half taper angle.

### 3.3. First Results

After coarse positioning of the pillars under measurement with the help of the auxiliary microscope, as shown in Figure 6, the side wall of the pillars can be scanned by the MEMS-SPM, so as to determine the central position of the pillar along its circumference and height.

#### 3.3.1. Surface Profiling of Nanopillars

**Figure 7:**
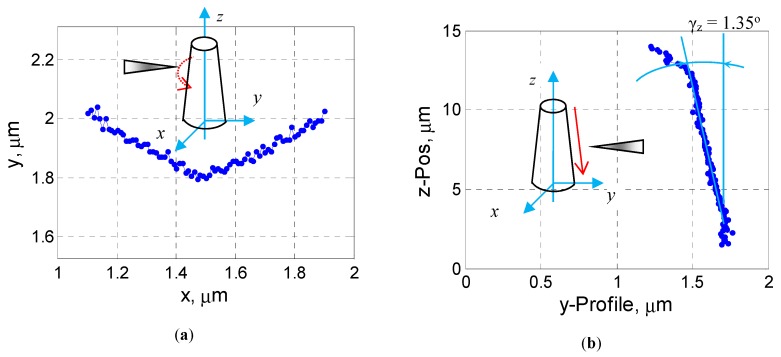
Surface scanning of a nanopillar in horizontal and vertical direction using the MEMS-SPM with a glued diamond probe: (**a**) Circumferential line profile of the pillar No. 1 scanned by the MEMS-SPM, from which a center position of *x* = 1.45 µm ± 0.05 µm on the pillar can be determined; (**b**) a line profile of the pillar along the *z*-axis (axial direction) at the center position *x* = 1.45 µm.

Figure 7a illustrates the measured profile of the pillar No. 1 along its cross-sectional circumference shown in Figure 6b at *z* = 2.0 µm. Of course, here, the measured line profile, which confirms the roundness deviation visible in Figure 5, is convolved with the topography of the AFM tip in use. The typical axial profile of the fabricated nanopillars is depicted in Figure 7b, and the pillar sidewall tilting angle relative to the vertical scanning axis amounts to *γ_z_* = 1.35°, coinciding well with the SEM image evaluation.

#### 3.3.2. Nano-Bending Testing

A series of nano-bending tests along the axial direction of the pillars have been performed, once the spatial position of a pillar had been measured.

At any given (*x*, *z*)-position, the pillar under test is firstly engaged with the diamond AFM probe by the piezo-positioning stage, and then lifted ca. 300 nm away from the probe. A passive nano-bending measurement is then followed: The pillar under test is moved incrementally to the diamond probe with a step interval of 5 nm, the in-plane deflection of the MEMS, and therefore the reaction force of the pillar during deflection, is measured simultaneously. Typically, the loading, holding, and unloading durations are 30 s, 10 s, and 30 s, respectively.

**Figure 8:**
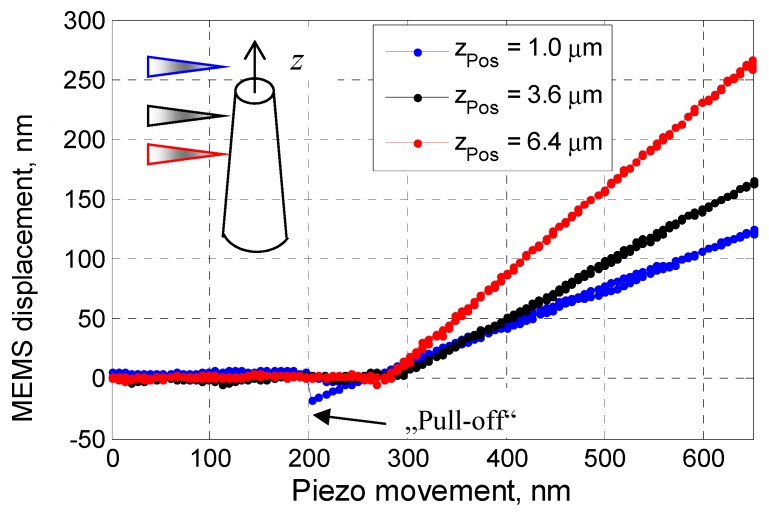
Bending tests of a nanopillar at different Δ*z*-positions. From the slope of the curves (*S_b_*(*z*)) the bending stiffness of the pillars *k_b_*(*z*) can be determined.

As examples, Figure 8 details several typical measurement curves obtained for the pillar measured in Figure 7. Since the pillar’s bending deflection in measurement is much smaller than the pillar height, the bending curves after contact are perfectly linear. Suppose that the slope of the bending curve at a given *z*-position be *S_b_*(*z*), the bending stiffness of the pillar at this *z*-position *k_b_*(*z*) can be calculated as follows:

(1) $$k_{b}\left(z\right)=k_{\mathrm{MEMS}}\cdot \frac{S_{b}\left(z\right)}{1-S_{b}\left(z\right)}.$$

It is clear that the bending stiffness of the pillar at the position *z*_pos_ = 3.6 µm should be much smaller than that at *z*_pos_ = 6.4 µm; therefore, for the same piezo movement after contact, the measured MEMS deformation at *z*_pos_ = 3.6 µm is evidently smaller than that at *z*_pos_ = 6.4 µm.

The measurement curve at the position *z*_pos_ = 1.0 µm demonstrates an evident “pull-off” effect, indicating that the diamond probe tip at this position is already vertically higher than the pillar’s top surface.

#### 3.3.3. Measurement Data Evaluation for Tapered Nanopillars

**Figure 9:**
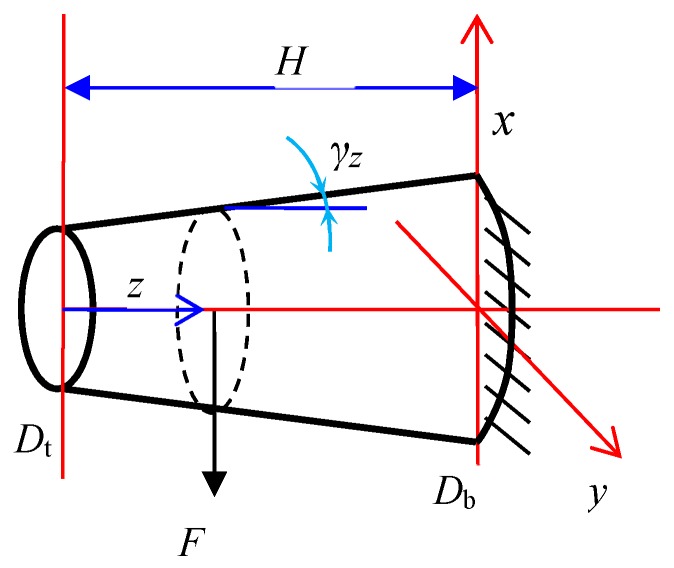
Schematic of a tapered rod defining the parameters for calculating its bending stiffness.

In this manuscript, the silicon nanopillars under measurement demonstrate clearly tapered 3D geometry. Under a given testing force *F* at the position Δ*z* (away from the top surface), the bending deflection of a tapered cylindrical rod made of isotropic materials with a length *L* at this position can be calculated as follows [21]:

(2) $$\delta_{x}=\frac{F{\left(H-z\right)}^{3}}{3EI_{z}}\cdot \frac{1}{r{\left(z\right)}^{3}},$$

where the local tapering ratio *r*(*z*) = *D_b_*/*D_z_* = [*D_t_* + 2*H* × tan(*γ_z_*)]/[*D_t_* + 2×*z*×tan(*γ_z_*)], *E* is the elastic modulus of the rod material, and *I_z_* is the moment of inertia at this position, which can be deduced as follows (Figure 9 shows a schematic of a pillar clamped at its right end with a transversal force *F* applied at different *z* positions):

(3) $$I_{z}=I_{t}{\left[1+\left(r\left(z\right)-1\right)\cdot \frac{z}{H}\right]}^{4}=\frac{\pi }{64}D_{t}^{4}{\left[1+\left(r\left(z\right)-1\right)\cdot \frac{z}{H}\right]}^{4}.$$

In the case that the nanopillars are made of linear orthotropic materials like single-crystal silicon, it is interesting to point out that the elastic modulus *E* in Equation (3) actually corresponds to the *E_z_* of anisotropic materials, i.e., elastic modulus perpendicular to the *x*-*y* plane (wafer plane).

Finally, the bending stiffness of a tapered silicon rod at its axial position z with respect to the top surface can be obtained as:

(4) $$k_{b}\left(z\right)=3E_{z}I_{z}r{\left(z\right)}^{3}\cdot \frac{1}{{\left(H-z\right)}^{3}}.$$

Especially, the bending stiffness of the tapered pillar at its top surface (Δ*z* = 0) *k_bt_* amounts to:

(5) $$k_{bt}=\frac{3\pi }{64}E_{z}\frac{D_{t}D_{b}^{2}}{H^{3}}.$$

**Figure 10:**
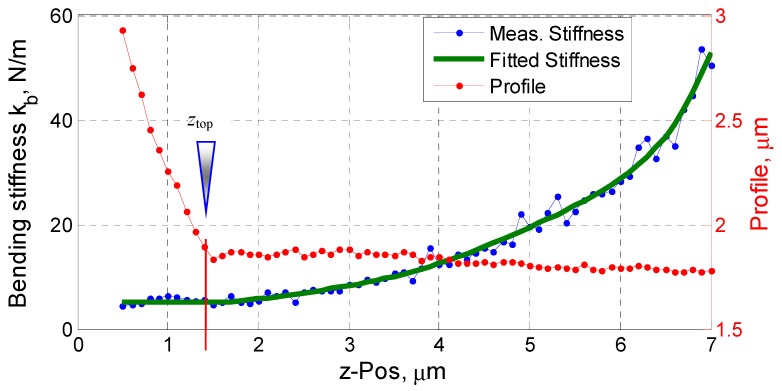
Measured profile and the bending stiffness of the pillar with respect to its axial positions.

Theoretically, it is possible to obtain the pillar’s bending stiffness from Equation (5) with a single bending measurement at the axial position of a pillar’s top surface. However, we have found it difficult to precisely determine the real axial position of a measurement. A series of bending tests along the pillar’s axis have been performed. As an example, Figure 10 depicts the profile and the measured bending stiffness of a pillar (shown in Figure 8) along its axial axis, and the pillar top surface is found at Δ*z*_top_ = 1.50 ± 0.05 µm.

To estimate the bending stiffness of the silicon pillar with better accuracy, the measured stiffnesses of the pillar at different *z*-positions have been fitted into a polynomial model, as illustrated in Figure 10 by the green curve. The measured bending stiffness of the pillar No. 1 at its top surface can be obtained from the fitted model. A series of bending measurements have been performed; finally, the experimental bending stiffness of the pillar No. 1 is found to be *k_b_* = 5.4 ± 0.2 N/m. Similar test procedure and data evaluation have been done also to the pillar No. 4. The measurement results are listed in Table 2.

In comparison, the experimental measurement results, finite element simulation results, and the analytical estimation by Equation (5) with the pillar parameters in Table 1 are listed in Table 2.

It can be seen from Table 2 that the measured bending stiffnesses of the two pillars at their top surfaces have slight differences (i.e., less than 2%), due to the shape imperfection caused by fabrication. The analytical and FEM results for the pillar No. 4 coincide quite well with the corresponding experimental value, since this pillar has nearly negligible shape imperfection (as shown in Figure 5d), and therefore can be well modelled by an ideal tapered cylinder for analytical and numerical analysis.

However, an evident shape imperfection appears for the pillar No. 1, due to the imperfect nanofabrication, as revealed in Figure 5c,d. This pillar can actually no longer be modelled by an ideal tapered cylinder, which will yield large deviation for analytical and numerical calculation of the bending stiffness, as detailed in Table 2.

#### 3.3.4. Measurement Uncertainty Estimation and Discussion

**Figure 11:**
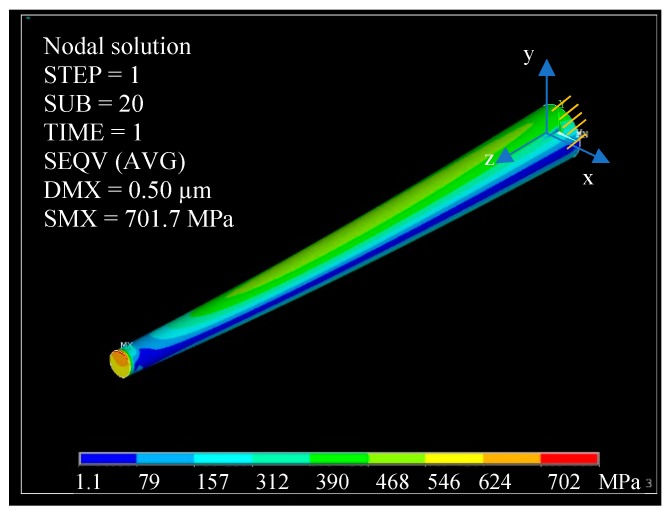
FEM simulation of the pillar No. 1 under maximum deflection (DMX is the maximum deflection of the pillar, and SMX is the maximum stress): The tapered pillar’s bottom surface in x-y plane is fixed. For the FE simulation of silicon pillars, the following anisotropic material constants were employed: *E_x_* = *E_y_* = 169 GPa, *E_z_* = 130 GPa, *G_xz_* = *G_yz_* = 80 GPa, *G_xy_* = 51 GPa, *ν_xy_* = 0.062, *ν_xz_* = *ν_yz_* = 0.28.

The maximum bending force *F*_max_ applied during the measurements illustrated in Section 3.3.3 is not larger than 4.0 µN. The FE simulation shown in Figure 11 reveals that the peak stress within the pillar No.1 with *h*_max_ = 500 nm amounts to about 700 MPa, which is far smaller than the yield strength of single crystal silicon, which is approximately 7 GPa. As a result, all the measurements in Section 3.3.3 should be performed within the elastic deformation range of the pillars.

Since the AFM probe tip radius *R*_tip_ ≈ 100 nm is usually much smaller than the pillar curvature, the maximum tip–surface penetration depth *h*_max_ can therefore be estimated by the typical elastic Hertzian contact:

(6) $$d_{\max}={\left(\frac{F_{\max}}{\frac{4}{3}E^{*}\cdot R_{\mathrm{tip}}{}^{1/2}}\right)}^{\frac{2}{3}}\leq 1.4\;\mathrm{nm},$$

where E* is the indentation modulus of silicon. Obviously, the pillar sidewall deformation (≤1.4 nm) during the nano-bending measurements is negligible compared to the pillar in-plane deflection and will therefore be omitted in the following uncertainty analysis.

The relative measurement uncertainty of the nano-bending method for the determination of the pillar’s bending stiffness can be deduced from Equation (1), as follows:

(7) $$\frac{u_{C}^{2}\left(k_{b}\right)}{k_{b}^{2}}=\frac{u^{2}\left(k_{\mathrm{MEMS}}\right)}{k_{\mathrm{MEMS}}^{2}}+\frac{u^{2}\left(S_{b}\right)}{{\left[S_{b}\left(1-S_{b}\right)\right]}^{2}}.$$

The uncertainty of the calibrated MEMS stiffness is *U*(*k*_MEMS_) = 4% [17]. The standard uncertainty for the determination of the curve slope *S_b_* is estimated at *u*(*S_b_*) ≈ 0.5%.

The second item *u*^2^(*S_b_*)/[*S_b_*(1 − *S_b_*)]^2^ in Equation (7) is dependent on the ratio of the pillar’s bending stiffness *k_b_* to the MEMS suspending stiffness *k*_MEMS_. In practice, the bending stiffness of the pillars under measurement *k_b_* is limited to *k_b_*/*k*_MEMS_ ∈ [0.1, 10]; the nondimensional variable *S_b_* will then range from 0.09 (for very soft pillars) to 0.9 (for hard pillars). Obviously, this uncertainty item will reach its minimum when *S_b_* = 0.5.

Finally, it can be concluded that this nano-bending measurement system will have the lowest measurement uncertainty of U(*k_b_*) = 4% (*k* = 2), when the pillars under measurement have the bending stiffness very close to the MEMS stiffness, i.e., *k_b_*/*k*_MEMS_ → 1. For very soft pillar (*k_b_*/*k*_MEMS_ → 0.1) or very stiff pillars (*k_b_*/*k*_MEMS_ → 10), the uncertainty *U*(*k_b_*) will approach 12% (*k* = 2).

## 4. Summary and Outlook

A MEMS scanning probe microscope with glued diamond AFM probe has been developed for the quantitative measurement of the bending stiffness of vertically aligned micro- and nanopillars with high aspect ratios. The MEMS-SPM features a force resolution better than 3 nN and an in-plane displacement sensing resolution of 0.2 nm, respectively. An experimental setup using an MEMS-SPM prototype has been built to experimentally determine the bending stiffness of silicon nanopillars with an average aspect ratio (height-to-diameter ratio) of 10.6.

Firstly, experimental results indicate that the prototype is able to determine the bending stiffness of vertically aligned pillars with an uncertainty better than 5%, when the pillars’ stiffness is close to the MEMS suspending stiffness. Furthermore, in comparison with an analytical approach for the calculation of the pillar bending stiffness, in which the geometrical dimensions of nanopillars have to be determined separately and very precisely, this experimental approach is much more rapid, reliable, and convenient.

Once the 3D geometry of nanopillars under measurement can be carefully and precisely modelled, this nano-bending measurement system can also be utilized to quantitatively determine the mechanical properties of pillar-like nano-objects at the nano-scale, which is one of the foci of our future research.

## Figures and Tables

**Table 1:** Geometrical dimensions of the tapered silicon pillars determined by SEM.

Dimensions	Pillar No. 1	Pillar No. 4
Top Diameter *D_t_*, nm	540 ± 15	552 ± 15
Height *H*, µm	11.83 ± 0.12	
Taper Angle (2*γ_z_*), degree	2.4 ± 0.2	1.9 ± 0.2
Bottom Diameter	1130 ± 84	944 ± 84

**Table 2:** The bending stiffness of nanopillars determined by experimental investigations and analytical calculation.

Bending Stiffness *k_bt_*, N/m	Pillar No. 1	Pillar No. 4
Nominal Value	6.8	
Experimental Measurements	5.4 ± 0.2	5.3 ± 0.1
Analytical Estimation	9.01	5.37
FEM Simulation	9.07	5.43
